# Impact of the angiotensin receptor-neprilysin inhibitor on chronic heart failure due to adult congenital heart disease: A systematic review and meta-analysis

**DOI:** 10.1016/j.jhlto.2025.100230

**Published:** 2025-02-18

**Authors:** Bibhuti B. Das, Shriprasad Deshpande, Lazaros Nikolaidis, Jianli Niu

**Affiliations:** a University of Mississippi Medical Center, Jackson, Mississippi; b Children’s National Hospital, The George Washington University, Washington DC, Washington; c Baylor Scott & White Medical Center, Temple, Texas; d Memorial Healthcare System, Hollywood, Florida

**Keywords:** ACHD, heart failure, sacubitril/valsartan, angiotensin receptor-neprilysin inhibitor, pharmacotherapy for ACHD heart failure

## Abstract

**Background:**

Heart failure (HF) is a significant complication in adults with congenital heart disease (ACHD), often requiring advanced therapeutic strategies. Angiotensin receptor-neprilysin inhibitors (ARNIs) have emerged as a promising alternative to angiotensin-converting enzyme inhibitors (ACEIs) and angiotensin receptor blockers (ARBs) in HF management. However, their safety and efficacy in ACHD-related HF remain unclear. This systematic review and meta-analysis aim to evaluate the impact of ARNIs on functional and safety outcomes in this unique patient population.

**Methods:**

We conducted a systematic review and meta-analysis of published studies assessing the use of ARNIs in ACHD patients with HF, comparing them to ACEIs/ARBs. The primary outcome was the change in New York Heart Association (NYHA) functional class (FC). Additionally, we assessed the safety profile of ARNIs in this population.

**Results:**

Our meta-analysis included 14 studies encompassing 305 patients. Substituting ACEIs/ARBs with ARNIs significantly improved the NYHA functional class (log odds ratio [log OR] 0.67, 95% CI 0.15–1.19; *p* = 0.01). ARNI therapy was associated with a notable reduction in systolic blood pressure (mean difference [MD] -0.49, 95% CI -0.70 to -0.29, *p* < 0.001) and an increase in serum creatinine levels (MD 0.30, 95% CI 0.10–0.49, *p* < 0.001). However, no significant change in serum potassium levels was observed (MD 0.00, 95% CI -0.61–0.61, *p* = 0.99).

**Conclusions:**

The addition of ARNIs to standard HF therapy may enhance functional outcomes in ACHD patients. However, the increased risk of hypotension and elevated serum creatinine levels necessitates careful monitoring. Further research is essential to better define the role of ARNIs in managing ACHD-related HF.

**Registration:**

URL: https://www.crd.york.ac.uk/prospero; Unique identifier: CRD42024591442.

## Background

Congenital heart disease (CHD) is present in approximately 1% of live births. With advancements in medical and surgical care, survival rates have significantly improved, allowing over 90% of individuals born with CHD to reach adulthood.^1^, ^2^ Adults with CHD (ACHD) face numerous challenges, particularly chronic heart failure (HF), which substantially affects both morbidity and mortality.^3^ It is estimated that 1 in 15 patients with ACHD will experience HF before the age of 42, a risk that is 106 times higher than that of the population with non-ACHD.^4^ As the number of patients with ACHD increases, the burden on health care systems due to HF management is expected to increase correspondingly.^5^, ^6^ The management of HF in patients with ACHD is intricate and has not been extensively studied. Previous studies recognized this deficiency, stating that despite the profound clinical implications of HF in ACHD, data supporting treatment recommendations—including established HF medications such as angiotensin-converting enzyme inhibitors (ACEIs), angiotensin receptor blockers (ARBs), beta-blockers (BBs), and mineralocorticoid receptor antagonists (MRAs)—are sparse for this patient group or ineffective.^7^, ^8^ In addition to drug therapy, addressing HF in patients with ACHD often requires transcatheter, surgical, or electrophysiological interventions to improve hemodynamic abnormalities temporarily but do not change the HF in the long term.

In recent years, the advent of angiotensin receptor-neprilysin inhibitor (ARNI) has revolutionized HF treatment in patients with non-ACHD, offering benefits beyond those provided by ACEIs or ARBs. Guideline-derived medical therapy now recommends ARNI/ACEIs/ARBs, BBs, MRAs, and sodium-glucose cotransporter-2 (SGLT-2) inhibitors for HF with a reduced ejection fraction (HFrEF) in the population with non-ACHD.^9^, ^10^ However, there remains a substantial gap in our knowledge regarding the role of ARNI in managing HF among patients with ACHD, as their safety and efficacy have yet to be thoroughly validated in this group. Given the potential shared pathophysiological mechanisms of HF in both patients with ACHD and non-ACHD, ARNI could offer similar benefits. This systematic review and meta-analysis aimed to elucidate the safety and effectiveness of ARNI therapy as a substitute for ACEIs/ARBs in the management of HF in patients with ACHD.

## Methods

### Search strategy and study selection

A comprehensive literature search was performed across the PubMed, Scopus, and Embase databases, which captured publications up to April 2024. Keywords included “heart failure in congenital heart disease,” “sacubitril-valsartan,” “ARNI,” “angiotensin neprilysin inhibitor,” “adult congenital heart disease,” and specific terms related to HF in various cardiac anatomies and guidelines. The selection process involved (1) identifying records through database searches, (2) removing duplicates, (3) screening abstracts, (4) assessing full-text articles for eligibility, and (5) final study inclusion. Discrepancies in study selection were resolved by a third reviewer (J.N.) following independent screening by 2 authors (B.D. and SD). We adhered to the Meta-Analysis of Observational Studies in Epidemiology guidelines during all stages of design, implementation, and reporting.^11^ This included the Preferred Reporting Items for Systematic Reviews and Meta-Analyses (PRISMA)^12^ and the Meta-Analysis of Observational Studies in Epidemiology Checklist. Our study protocol was registered on PROSPERO (International Prospective Register of Systematic Reviews; CRD42024591442).

### Inclusion and exclusion criteria

The decision to include prospective and retrospective clinical trials, case series, and reports on patients with ACHD with chronic HFrEF aged ≥18 years receiving standard HF therapy (BB, ACEI/ARB, and/or MRA) with the ARNI, introduced as a replacement, was based on the need to gather comprehensive data on the use of ARNI in this specific patient population. Studies on patients with decompensated HF and heart failure with preserved ejection fraction were excluded due to their complex definitions in the population with ACHD, which could introduce confounding factors and make the results less applicable to the intended patient group.

### HF severity assessment

The studies reported HF severity based on the left ventricular (LV) ejection fraction: <30% as severe, 30% to 40% as moderate, and >40% as mild. For systemic right ventricular (sRV) conditions, such as in congenitally corrected transposition of the great arteries (CCTGA), dextrotransposition of the great arteries (D-TGA) post-Glenn or Mustard operations, and hypoplastic left heart syndrome following Fontan surgery, HF severity was assessed using RV fractional area change and tricuspid annular plane systolic excursion as reported in the included studies. Similarly, post-ARNI changes in ventricular function were documented via echocardiographic parameters, including the LV ejection fraction and global longitudinal strain for the systemic LV (sLV) and the RV fractional area change and tricuspid annular plane systolic excursion for the sRV.

### Data extraction

Data on the New York Heart Association (NYHA) functional class (FC), 6-minute walk distance, and cardiopulmonary exercise test (CPET) results were collected to evaluate improvements in exercise capacity. B-type natriuretic peptide (BNP) or N-terminal pro-B-type natriuretic peptide (NT-proBNP) levels were recorded before and after ARNI treatment. For this study, we calculated the average daily dose of ARNI by dividing the total ARNI dose received during all study visits by the total days in the study, following the approach used in the Prospective Study of Biomarkers, Symptom Improvement, and Ventricular Remodeling During Sacubitril/Valsartan Therapy for Heart Failure trial.^13^

### Risk of bias (quality) assessment

Two independent reviewers (B.D. and S.D.) evaluated the risk of bias and the quality of the included studies using the Newcastle-Ottawa scale for observational research.^14^ Any disagreements were resolved through discussion with a third reviewer (J.N.). We assessed 3 criteria: cohort selection (0-4 stars), comparability (0-2 stars), and outcomes (0-3 stars). Overall scores were categorized as follows: less than 5 stars indicated a high risk of bias, 5 to 7 stars represented some concerns, and more than 7 stars indicated a low risk of bias (Table 1).

**Table 1:** The Newcastle-Ottawa Scale Quality Assessment of the Included Studies

### Outcomes

The primary outcome, an improvement in NYHA FC in patients with ACHD HF after replacing ACEIs or ARBs with ARNI, is of particular interest as it directly measures the impact of ARNI therapy on the patient's functional capacity. Additionally, we assessed the safety and tolerability of ARNI by studying adverse effects, including hypotension (as reported in each article), renal dysfunction (defined by either increased serum creatinine [Cr] or decreased estimated glomerular filtration rate), and hyperkalemia (increased potassium [K+] levels compared with values before ARNI initiation). We documented the number of patients who discontinued therapy for adverse effects only, and we did not include those lost to follow-up, those who underwent heart transplantation, or those who died.

### Statistical analysis

Data from the included studies were analyzed via IBM SPSS Statistics version 29. The impact of the ARNI on the NYHA FC was assessed via pooled log odds ratios (log [ORs]) with 95% confidence intervals (CIs). To quantitatively evaluate the effects of the ARNI on key outcomes such as NT-proBNP levels, systolic blood pressure, and serum Cr levels, a pooled standardized mean difference (MD) with 95% CI was calculated. We utilized the Higgins I^2^ statistics to compute between-study heterogeneity calculations. Low heterogeneity and high heterogeneity are defined by I^2^ <25% and >75%, respectively.^15^ A random-effects model was employed for all pooled analyses due to the anticipated clinical heterogeneity. The tau-squared (τ^2^) statistic within the random-effects model framework estimated the between-study variance. Statistical significance was determined by a 2-tailed test with a *p*-value threshold of <0.05.

## Results

### Literature search and study characteristics

**Figure 1:**
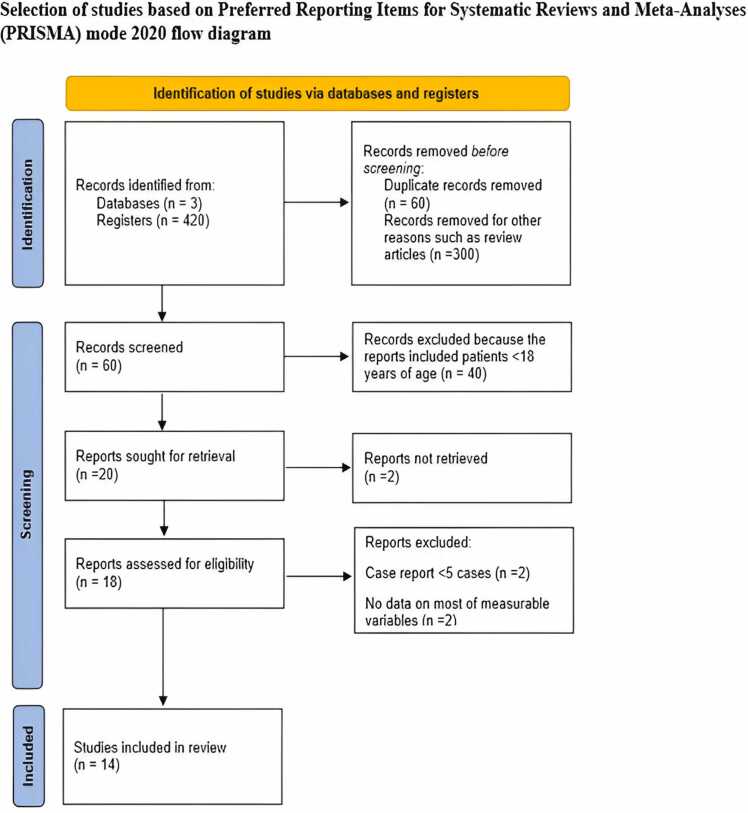
Selection of studies based on the Preferred Reporting Items for Systematic Reviews and Meta-Analyses (PRISMA) model.

The literature search was conducted with meticulous attention to detail, initially identifying 420 publications. The comprehensive process of the literature search is illustrated in the PRISMA flowchart (Figure 1). Ultimately, 14 observational studies,^16^, ^17^, ^18^, ^19^, ^20^, ^21^, ^22^, ^23^, ^24^, ^25^, ^26^, ^27^, ^28^, ^29^ encompassing 305 patients, were included in both quantitative and qualitative analyses.

### Patient categorization

The age range was 25 to 84 years (median: 42 years). The study designs were predominantly retrospective (72%), with the remainder being prospective (14%), randomized controlled trials (7%), and case series (7%). The cohort was 65% male, with a median follow-up of 235 days (180-361 days). Patients were classified by morphologic ventricle type: sRV in 212 (70%) with biventricular circulation (65% had D-TGA status post atrial switch, and 35% had CCTGA), sLV in 84 (27%) with biventricular circulation, and only 9 (3%) with Fontan circulation and an unknown ventricle type. We also classified the entire cohort based on anatomic defect and physiological status, adhering to the 2018 American Heart Association/American College of Cardiology guidelines for ACHD classification,^30^ into 12 categories (Table 2).

**Table 2:** Classification of Patients With ACHD Based on Anatomic Defect and Physiological State

Abbreviation: ACHD, adult congenital heart disease.

### Drug therapy and implantable devices for HF

**Figure 2:**
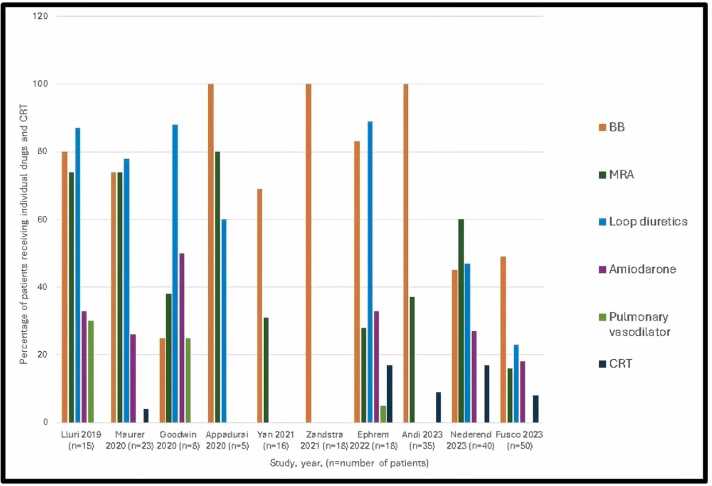
Bar diagram summarizing the percentage of patients receiving each drug for heart failure in each study before initiating angiotensin receptor-neprilysin inhibitor (ARNI). BB, beta-blockers; MRS, mineralocorticoid receptor antagonist.

Before ARNI initiation, all patients were on ACEIs/ARBs therapy with a 36-hour washout period. Background HF therapies included BBs (69.2%), MRAs (27.2%), loop diuretics (42.1%), antiarrhythmic drugs (18%), digoxin (4.8%), and pulmonary vasodilators (5.2%) (Figure 2). The median daily dose of ARNI was 173 mg (interquartile range: 128-190 mg), with a median treatment duration of 6 months (range: 3 months to 1 year). Dose adjustments were based on patient tolerance and blood pressure. With respect to implantable devices, 32% had pacemakers, 9% had implantable cardioverter-defibrillators, and 8% had cardiac resynchronization therapy (CRT) devices.

### Key outcomes

**Figure 3:**
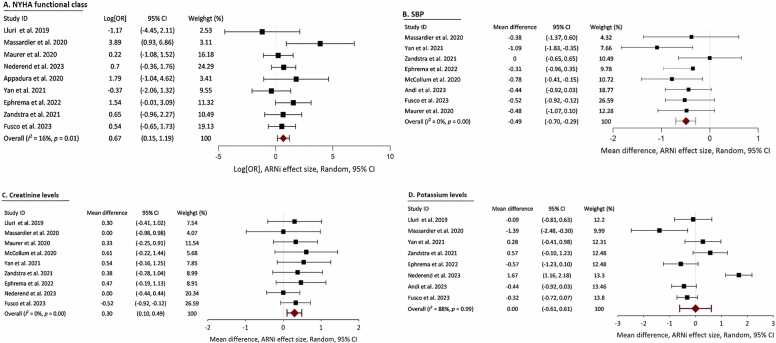
Meta-analysis evaluating the impact of angiotensin receptor-neprilysin inhibitor (ARNI) therapy in adults with congenital heart disease and chronic heart failure. (A) Significant improvement in New York Heart Association (NYHA) functional class, reflecting enhanced clinical status and symptom relief. (B) Notable reduction in systolic blood pressure, underscoring the importance of personalized hemodynamic monitoring. (C) Modest increase in serum creatinine levels following the transition from angiotensin-converting enzyme inhibitors or angiotensin receptor blockers to ARNI, highlighting the need for renal function monitoring in this population. (D) Stable serum potassium levels, demonstrating ARNI's favorable safety profile with respect to electrolyte balance. CI, confidence intervals; OR, odds ratios; SBP, systolic blood pressure.

The impacts of the ARNI on NYHA FC, ventricular function, and natriuretic peptide levels reported in each article are summarized in Table 3. ARNI therapy led to a significant improvement in NYHA FC (log [OR] 0.67, 95% CI 0.15-1.19, *p* = 0.01), with low heterogeneity (I^2^ = 16%) (Figure 3A). Ventricular function, as measured by the ejection fraction, did not significantly improve in 2 studies (MD 0.37, 95% CI −0.45 to 1.2, *p* = 0.38), with high heterogeneity (I^2^ = 78%). Natriuretic peptide (BNP or NT-proBNP) levels did not significantly change (Pearson’s chi-square test *p* = 0.67). Eight studies reported changes in blood pressure. One hundred and ten patients (36%) experienced a significant decrease in systolic blood pressure (MD −0.49, 95% CI −0.70 to 0.29, *p* < 0.001), with low heterogeneity (I^2^ 0%) (Figure 3B). Similarly, the ARNI was associated with a significant reduction in diastolic blood pressure (MD −0.31, 95% CI −0.56 to 0.05, *p* = 0.02), consistent across studies (I^2^ = 0%). Nine studies reported the impact of the ARNI on Cr levels. ARNI was associated with significantly elevated Cr levels compared with baseline (MD 0.30, 95% CI 0.10-0.49, *p* = 0.00), with low heterogeneity (I^2^ = 0%) (Figure 3C). Eight studies evaluated changes in the serum K+ concentration after adding the ARNI to therapy. No significant change was observed in the serum K+ concentration (MD 0.00, 95% CI −0.61 to 0.61, *p* = 0.99), with low heterogeneity (I^2^ = 0%) after the addition of the ARNI (Figure 3D). A few studies^20^, ^21^, ^22^, ^27^, ^29^ reported improvements in either 6-minute walk distance or CPET using variable parameters, precluding us from conducting a meaningful meta-analysis for these outcomes.

**Table 3:** Main Outcomes After ARNI Added to Combined HF Therapy in Patients With ACHD

		NYHA FC improvement, N (%)	Improvement in ventricular function by echocardiogram	BNP decreased or no change	NT-proBNP decreased or no change
Study	N				
Lluri et al^16^	15	4 (26%)	NR	NR	NR
Massardier et al^17^	8	6 (75%)	NS	NR	NR
Maurer et al^18^	23	NS	NS	NR	NS
Goodwin et al^19^^,^^a^	8	NS	NR	Decreased	NR
McCollum et al^20^^,^^a^	21	12(57%)	NR	NS	NR
Appadurai et al^21^	5	4 (80%)	Improved	NS	NR
Zandstra et al^22^	18	5(28%)	Improved	NR	Decreased
Yan et al^23^	16	2 (10%)	Improved	NS	NR
Ephrem et al^24^	18	12 (66%)	NS	NS	NR
Andi et al^25^	35	21 (60%)	Improved	NR	Decreased
Gupta et al^26^^,^^a^	35	16 (45.7) %	NS	NS	NR
Nederend et al^27^	40	5 (12.5%)	Yes	NR	Decreased
Sanfilippo et al^28^^,^^a^	13	9 (69%)	NS	NR	NR
Fusco et al^29^	50	19 (38%)	Improved	NR	Decreased

Abreviations: ACHD, adult congenital heart disease; ARNI, angiotensin neprilysin inhibitor; BNP, B-type natriuretic peptide; FC, functional class; HF, heart failure; NR, not recorded; NS, statistically no significant change as reported in each study; NYHA, New York Heart Association.

a Abstracts: limited data.

### Safety profile

The thorough safety assessments focused on hypotension, renal dysfunction, and hyperkalemia were performed as reported in each study and are summarized in Table 4. Eighteen (6%) patients discontinued ARNI because of adverse effects.

**Table 4:** Side-Effects of ARNI After Added to Combined HF Therapy in Patients With ACHD

Study	N	Statistically significant decrease in BP compared to Baseline, *p*-value	Statistically significant increase in serum creatinine compared to baseline, *p*-value	Statistically significant increase in serum potassium compared to baseline, *p*-value	Discontinuation of ARNI due to adverse effects only N (%)
Lluri et al^16^	15	NS	NS, *p* = 0.22	NS, *p* = 0.68	1 (7%)
Massardier et al^17^	8	NS, *p* = 0.23	NS, *p* = 0.52	NS, *p* = 0.11	0
Maurer et al^18^	23	Yes, *p* = 0.01	Increased, *p* = 0.0002	NR	4 (17%)
Goodwin et al^19^^,^^a^	8	NR	NS	NS	0
McCollum et al^20^^,^^a^	21	Yes, *p* = 0.009	NS, *p* = 0.87	NS	2 (10%)
Appadurai et al^21^	5	NS	NS	NS	0
Zandstra et al^22^	18	NS	NS	Increased, *p* = 0.01	0
Yan et al^23^	16	Yes, *p* = 0.0016	Increased, *p* = 0.016	NS	0
Ephrem et al^24^	18	NS	NS	NS	0
Andi et al^25^	35	NS	NS	NS	0
Gupta et al^26^^,^^a^	35	NS	NS	NS	3 (9%)
Nederend et al^27^	40	NS	NS	NS	5 (13%)
Sanfilippo et al^28^^,^^a^	13	NS	NS	NS	1 (8%)
Fusco et al^29^	50	Yes, *p* < 0.01	NS	NS	2 (4%)

Abreviations: ACHD, adult congenital heart disease; ARNI, angiotensin neprilysin inhibitor; BP, blood pressure; HF, heart failure; NR, not recorded; NS, no significant change.

*p*-value in each study is as recorded in that study.

a Abstracts: limited data.

## Discussion

Extensive research has already explored the established benefits of ARNI in treating HFrEF in adults without CHD. Recently, a nationwide retrospective study from the German National Register for CHD showed that 38.5% of patients with ACHD with sRV suffering from ventricular dysfunction, systemic atrioventricular valve regurgitation, and arrhythmias were not prescribed any HF medications.^31^ In this systematic review and meta-analysis, ARNI is superior to ACEI/ARB therapy in patients with ACHD HF. Our key findings include that the ARNI improved NYHA FC, indicating enhanced functional status in ACHD HF patients. Notably, there was no significant improvement in ventricular function or decrease in BNP/NT-proBNP levels in patients with ACHD HF for whom data were available. These results were consistent across sRV, sLV, single ventricle physiology, and complex CHD from stages A to D, with varying degrees of ventricular dysfunction.

The median age of HF in patients with ACHD (42 years) was much younger than the mean age of the recruited population in the PARADIGM-HF trial (63.8 ± 11.5 years). PARADIGM-HF only enrolled patients with LVEF above 40%. Additionally, 60% of patients in the PARADIGM-HF trial had ischemic heart disease as the primary etiology of HFrEF.^32^ In our meta-analysis, most ACHD HF patients did not reach the target ARNI dose used in the PARADIGM-HF trial,^32^ where the target daily dose was 97 mg/103 mg of sacubitril-valsartan twice daily. The PARADIGM-HF trial compared 200 mg of ARNI twice daily to 10 mg of enalapril. It is important to note that the PARADIGM-HF trial did not include NYHA FC IV or patients with hypotension and arrhythmia. In our analysis, hypotension posed challenges for increasing the ARNI dose in ACHD HF patients.

The sacubitril component of ARNI inhibits neprilysin, also known as neutral endopeptidase, an enzyme responsible for the degradation of several natriuretic peptides, including BNP and NT-proBNP. This inhibition increases these endogenous vasoactive peptides' levels, enhancing their beneficial effects, such as vasodilation, natriuresis, and diuresis. On the other hand, the valsartan component of ARNI is an ARB that antagonizes the effects of angiotensin II, resulting in vasodilation and reduced aldosterone release. In addition, ARNI has demonstrated superiority over ACEIs and ARBs in patients with HF due to its enhanced effects on myocardial remodeling. Sacubitril and valsartan act synergistically to prevent cardiomyocyte cell death and matrix remodeling by preventing the breakdown of endogenous vasoactive peptides and inhibiting various subunits of guanine nucleotide-binding proteins.^33^, ^34^ Interestingly, these effects are only seen when the 2 drugs are combined instead of used separately. Combining these 2 drugs offers a synergistic effect that improves cardiac function and reduces hospitalization risk for HF patients without CHD. In rat models of cardiorenal syndrome, ARNI mitigates fibrosis, oxidative stress, mitochondrial damage, endothelial dysfunction, cardiac remodeling, and apoptosis in kidney and heart tissues.^35^, ^36^, ^37^, ^38^ Despite the well-established efficacy of ARNI in patients with non-ACHD HF, their integration into the treatment of patients with ACHD HF remains limited.^32^

Previous studies examining ACEIs or ARBs alone have yielded mixed results, especially in patients with ACHD HF.^39^, ^40^, ^41^, ^42^ In the ACHD HF cohort, 179 patients had data on implantable devices, including pacemakers (32%), implantable cardioverter-defibrillators (9%), and CRTs (8%). The prevalence of pacemaker use was more significant than that reported in the PARADIGM-HF trial,^43^ likely due to multiple past surgical procedures adversely affecting the cardiac conduction. However, patients with ACHD HF received CRT in 7.5% of the cases, such as patients with non-ACHD in the PARADIGM-HF trial.^43^

The impacts of ARNI on Cr and K+ levels and hypotension were the 3 most common adverse effects in the PARADIGM-HF trial.^43^ In our meta-analysis, hypotension and elevated serum Cr were observed in a significant percentage of patients with ACHD HF. Interestingly, there was no substantial change in the serum K+ level. Importantly, in a recent meta-analysis and systemic review in patients with HF, ARNI use was shown to be protective against renal impairment, with ARNI users having a lower risk of renal dysfunction and a higher estimated glomerular filtration rate without an increased risk of hyperkalemia compared with traditional inhibition of the renal aldosterone-angiotensin system.^44^ Undoubtedly, the small number of patients included in the meta-analysis may account for the spurious increase in the percentage of adverse effects in patients with ACHD HF. Despite these differences, we can safely assume that ARNI is tolerated in most patients with ACHD HF. The discontinuation rate in patients with ACHD HF was 6%, primarily due to hypotension and elevated Cr levels, whereas it was 10.7% in the PARADIGM-HF trial. This discontinuation rate may be further decreased by educating prescribers about acceptable elevation in Cr early during initiation and about persisting with these medications through this early period to derive long-term benefits.^45^, ^46^

Nederend et al^27^ showed that the beneficial effects of ARNI are more pronounced when therapy is continued for 3 years than when it is continued for 6 months. These findings suggest that ARNI can provide long-lasting benefits for the longitudinal control of HF in patients with ACHD. Recently, preliminary data from the prospective comparison of ARNI vs plAcebo in patients with congenital sYStemic Right Ventricular heart failure (PARACYS-RV) trial demonstrated that ARNI in patients with sRV dysfunction and NYHA class II and III symptoms is effective and is associated with favorable trends regarding improvement in total exercise duration and decreased NT-proBNP.^47^ The promising results from PARACYS-RV trial and encouraging observational studies^16^, ^17^, ^18^, ^19^, ^20^, ^21^, ^22^, ^23^, ^24^, ^25^, ^26^, ^27^, ^28^, ^29^ support the idea that ARNI could be effective in chronic HF due to ACHD. The PARACYS-RV was a prospective crossover trial. Crossover trials carry the advantage of removing intersubject variability from group comparisons since each subject serves as their own control. This maximizes the study power and statistical efficiency, desirable features in the study of ACHD in the HF population. However, PARACYS-RV crossover trial revealed more adverse effects on participating patients during the washout period when ARNI was switched back to ARB/ACEI. The study was terminated prematurely, but it is suggested that the washout periods should be longer to avoid the carryover effects of previous drugs in future studies. There are ongoing future studies from registry data, such as the Pathfinder-CHD Registry,^48^ ARTORIA-R,^49^ and ENTRUST ACHD-HF registry.^50^ Hopefully, these studies will better guide ARNI use in patients with ACHD in the future.

### Study limitations

Our meta-analysis has several limitations. First, there was a variability in the echocardiographic parameters used across studies to estimate HF severity, particularly for patients with sRV. Additionally, differences in ARNI dosages and durations, influenced by the spectrum of anatomic diagnoses and physiological states in patients with ACHD with HF, further contributed to heterogeneity. The diverse patient populations, recruitment characteristics, and limited availability of granular data (e.g., age at initial CHD repair, residual anatomic lesions) also introduced variability and inconsistencies. While some studies reported pairwise pre- and post-ARNI differences for quantifiable variables such as CPET and 6-minute walk distance, the lack of comprehensive data significantly hinders our understanding of ARNI’s effects on exercise performance. Future research addressing this gap is crucial to fully elucidating the benefits of ARNI therapy. Patients with CCTGA and D-TGA after atrial switch operations (e.g., Mustard or Senning) were analyzed together for sRV outcomes. However, these groups differ in their surgical histories, ages at HF development, and susceptibility to rhythm disturbances. Importantly, these patients represent distinct forms of ACHD compared to those with single ventricle physiology, where HF is often present from early life. The limited sample size of only 9 patients with Fontan circulation precludes generalizing ARNI use to this specific population with ACHD. Given the unique hemodynamics and long-term complications associated with Fontan physiology, such as Fontan-associated liver disease and protein-losing enteropathy, further research is essential to determine ARNI’s safety and efficacy in these patients. We were also unable to compare outcomes between sRV and sLV in patients with ACHD due to insufficient granular data in the included studies. However, most studies utilized control groups with similar ACHD characteristics, which partially mitigates this limitation. While our study evaluated ARNI in combination with conventional HFrEF therapies (e.g., BBs and MRAs), it did not explore the role of SGLT-2 inhibitors in patients with ACHD with HF. Future research on SGLT-2 inhibitors in this population is critical, as they have earned a class I recommendation for HFrEF treatment in patients with non-CHD according to American College of Cardilogy/American Heart Association and European Society of Cardiology guidelines.^9^, ^10^ However, like ARNI, the use of SGLT-2 inhibitors in patients with ACHD remains limited.^51^, ^52^ Whether combining the “four pillars” of HFrEF therapy (ARNI, BB, MRA, and SGLT-2 inhibitor) in patients with ACHD is as feasible, safe, and effective as in patients with acquired heart disease remains an important hypothesis for future investigation.

## Conclusions

Given the high rates of hospitalization and mortality among patients with ACHD due to worsening HF, ongoing efforts are essential to deepen our understanding of the underlying pathophysiological mechanisms and develop targeted therapies for this growing population. This meta-analysis demonstrates that ARNI therapy offers a therapeutic advantage by improving NYHA FC, potentially benefiting a broad spectrum of patients with ACHD with HF, including those with mild to severe ventricular dysfunction. The integration of ARNI into treatment regimens is associated with a favorable safety profile across patients with ACHD, from stages A to D HF. However, clinicians must remain vigilant about common adverse effects, such as hypotension and elevated serum Cr levels, which may hinder optimal dosing. This underscores the importance of careful dose titration and personalized medicine to address the unique needs of each patient.

In conclusion, ARNI represents a promising therapeutic option for patients with ACHD with HF, offering both clinical and functional benefits. However, its optimal use requires careful patient selection, monitoring, and further research to address existing knowledge gaps. By advancing our understanding of ARNI’s role in ACHD, we can move closer to improving outcomes and quality of life for this vulnerable population.

## Ethical approval

Not applicable.

## Disclosure statement

The authors declare that they have no conflicts of interest relevant to this article. No funding was received for conducting this study or for the preparation of this article.
